# A comparative analysis of deep learning architectures with data augmentation and multichannel input for locoregional breast cancer radiotherapy

**DOI:** 10.1002/acm2.70047

**Published:** 2025-02-20

**Authors:** Rosalie Klarenberg, Nienke L. M. Bakx, Coen W. Hurkmans

**Affiliations:** ^1^ Department of Radiation Oncology Catharina Hospital Eindhoven Eindhoven The Netherlands; ^2^ Department of Electrical Engineering and Department of Applied Physics and Science Education Technical University Eindhoven Eindhoven The Netherlands

**Keywords:** 2D and 3D, artificial intelligence, breast cancer, data augmentation, deep learning, dose prediction, input channels, radiotherapy

## Abstract

**Purpose:**

Studies on deep learning dose prediction increasingly focus on 3D models with multiple input channels and data augmentation, which increases the training time and thus also the environmental burden and hampers the ease of re‐training. Here we compare 2D and 3D U‐Net models with clinical accepted plans to evaluate the appropriateness of using less computationally heavy models.

**Methods:**

A 2D Attention U‐Net, a 2D HD U‐Net, and a 3D U‐Net were trained using 1 or 5 input channels with or without data augmentation and data from 89 locoregional breast cancer patients. Results were compared to clinically accepted plans. The significance of inclusion of more channels or data augmentation was compared to the base models and the HD U‐Net and Attention U‐Net were compared to their respective identically trained counterparts.

**Results:**

The Attention U‐Net reached fewest PTV clinical goals (28%, mostly due to a too high average breast PTV dose) and improved using significantly using five channels and augmentation (49%). The HD U‐Net already fulfilled 70% of the PTV goals, which did not improve much by adding more channels or augmentation. The 3D U‐Net with five channels and augmentation reached 76%, compared to 81% in the clinically accepted plans. The lower rates for the HD U‐Net compared to the 3D U‐Net and clinical plans were mainly caused by a lower PTVn1n2 D98%, which was still on average 93%. Organ‐at‐risk goals were met in most cases for all models. Training time was approximately 8 fold for the 3D model.

**Conclusions:**

Comparable results to a 3D U‐Net and clinical plans can be reached with a 2D HD U‐net for a dataset size commonly seen in clinical practice. The Attention U‐Net did profit from adding extra channels and data augmentation but did not reach the same level of accuracy as the other models.

## 1 INTRODUCTION

Radiotherapy treatment planning for locoregional treatment of breast cancer can be considered complex and is often time consuming and user dependent. The use of automation, particularly artificial intelligence (AI) and more specifically deep learning (DL) models, has been shown to enhance efficiency, ensure greater consistency, and reduce inter‐operator variability in treatment planning.^1^, ^2^

DL models have proven quite accurate and efficient in predicting radiation dose distributions for various cancer sites, including head and neck (H&N) tumors, cervical cancer, and breast cancer.^3^, ^4^, ^5^, ^6^ Most studies investigating dose prediction models focus on studying U‐Net shaped architectures, initially proposed by Ronneberger et al. for biomedical image segmentation.^7^ For example, Nikolov et al. employed this architecture for dose prediction for H&N cancer treatment plans.^3^ Nguyen et al. proposed the hierarchically densely connected (HD) U‐Net for predicting such dose distributions,^4^ and showed that this architecture is highly efficient and outperformed the standard U‐Net and DenseNet. Oktay et al. implemented Attention‐gated U‐shaped architectures (Attention U‐Nets) for image segmentation, which focus on certain structures and shapes and suppress irrelevant features.^8^ The Attention U‐Net has been employed by others to predict dose distributions for H&N treatment plans^9^, ^10^ and cervical brachytherapy.^11^

It is not straightforward to compare the outcomes of separate studies focusing on a specific network architecture and conclude which architecture is better. A few comparative studies on predicting dose distributions have been published. These concentrated on 3D networks, comparing U‐Net, Attention U‐Net and HD U‐Net.^12^, ^13^ Such 3D networks have a heavy computational demand which is not optimal as training costs more time, making it more difficult to adapt to changes in a treatment technique through retraining. Furthermore, such models cause a higher environmental burden than less computational demanding networks. The goal of our work was to see if a 2D Attention U‐Net or HD U‐Net could perform equally well as a commercially available 3D U‐Net model for left‐sided locally advanced breast cancer treatment plans. In addition, we studied the influence of performing data augmentation and the number of input channels on model performance.

## 2 MATERIALS AND METHODS

### 2.1 Patient data

Anonymized treatment planning data from 89 left‐sided locally advanced breast cancer patients was used in this study. For 41 patients the target volume included the breast and lymph node levels 1 and 2 and for the other 48 patients lymph node levels 1 to 4 were included. All patients were treated with 15 fractions IMRT with a prescribed dose of 4005 cGy. The treatments were delivered between 2018 and 2023 in the radiotherapy department at the Catharina Hospital in Eindhoven (The Netherlands). The dataset of each patient contains a CT scan of the patient, delineated organs‐at‐risk (OARs) and clinical target volumes (CTVs) according to ESTRO guidelines and a manually optimized radiation treatment plan.^14^ Planning target volumes (PTVs) were also included, which were expansions of the CTVs by 5 mm, limited to 5 mm below the skin. Separate PTVs were defined for the breast as the primary target (PTVp), lymph node levels 1 and 2 combined (PTVn1n2) and levels 3 and 4 combined (PTVn3n4). In the rest of this paper the combination of these PTVs will be referred to as PTV.

### 2.2 Model training

In total, 3 DL models were trained with different combinations of the number of input channels and with or without applying augmentation, as shown in Table 1. The three different DL architectures that were explored are a 2D hierarchically densely connected (HD) U‐Net,^5^ a 2D Attention U‐Net^10^ and a 3D U‐Net model.^15^

**TABLE 1:** Overview of trained DL architectures, number of input channels and data augmentation use.

DL architecture	Input	Data augmentation
2D Attention U‐Net	PTV	No
2D Attention U‐Net	PTV	Yes
2D Attention U‐Net	PTV, heart, lungs, humerus, and external body contour	No
2D Attention U‐Net	PTV, heart, lungs, humerus, and external body contour	Yes
2D HD U‐Net	PTV	No
2D HD U‐Net	PTV	Yes
2D HD U‐Net	PTV, heart, lungs, humerus, and external body contour	No
2D HD U‐Net	PTV, heart, lungs, humerus, and external body contour	Yes
3D U‐Net	PTV, heart, lungs, humerus, and external body contour	Yes

The HD U‐Net architecture used in this work was taken from the work of van de Sande et al., showing the potential for this model in locally advanced breast cancer.^5^ The Attention U‐Net utilizes an attention‐gating mechanism to highlight relevant activations during training, reducing the number of redundant features. We took the implementation of Osman and Tamam which successfully deployed this architecture for the dose prediction of H&N cancer treatment plans.^10^ Finally, a 3D U‐Net was trained using a training framework provided by the vendor of our treatment planning system (TPS) (RayStation, RaySearch Laboratories AB, Sweden). For all models, more information can be found in the Figure S1.

Each input channel only contained the Euclidean distance maps based on one segmentation: either the PTV (combination of primary PTV and lymph nodes) or one of the OARs or the external body contour. These distance maps contained positive values for voxels within the respective ROI, and negative values outside. These Euclidean distance maps were used to keep proximity information about the ROIs in the craniocaudal direction. The 2D data was stored as individual sliced datasets, and during training, slice selection was performed through Gaussian sampling. A batch size of 24 slices was used, consisting of 8 slices from 3 different patients. A more detailed description of the pre‐processing and sampling process is described by van de Sande et al.^5^ For the 3D U‐Net, it was chosen to use the original pre‐processing as implemented in the training framework of the TPS model. During this pre‐processing, the input was centred around the PTVp and the dose is normalized, with the prescribed dose set to 1. The input consisted of binary masks of the PTV and separate channels for each of the OARs.

For the 2D model, augmentation is applied with a likelihood of 50% for each slice. When augmentation is performed, three techniques are applied to the slice with different probabilities: affine transformation (*p* = 0.8), with a shift up to a maximum of 20% relative to image size, flipping (*p* = 0.8) and rotation (*p* = 0.3), with a random angle within the range of −45 to 45 degrees.

For the 3D U‐Net, a combination of four techniques is applied when augmentation is performed: rotation (range of −10 to 10 degrees), translation (maximum 7 voxels), scaling (maximum 10%) and sagittal flipping (*p* = 0.5).

All models were trained using 5‐fold cross validation. For training and validation, 80% of the patients were used, whereas the remaining 20% was used for model testing. These test patients were randomly sampled from the complete dataset, while ensuring an equal ratio of patients with node level 1–2 and node level 1–4 in both training and test set. The remainder of the dataset was then split into stratified folds, based on the PTV volumes of the patients and the same ratio of node level 1–2 and 1–4, ensuring a similar distribution of patient characteristics over the folds.

### 2.3 Prediction and evaluation

The final prediction for each patient was calculated as the mean of the predicted dose distributions of the 5 folds. All dose distributions, including the clinical, were scaled such that 98% of the PTVp volume received 95% of the prescribed dose for comparison. The clinical goals used in our clinic, based on the Dutch national consensus, were used to compare plan quality.^16^ Besides, several DVH parameters for both PTVs and OARs, such as average and maximum dose (D2%) were calculated (see Table S1). The Wilcoxon signed‐rank test was used to assess statistical significance between any of the dose parameters and the clinical plan, but also to compare for each 2D architecture the effect of using extra input channels and/or data augmentation, compared to only using the PTV and no data augmentation. Finally, statistical significance was assessed for each of the counterparts of the two 2D DL architectures. The time to train the different models was also registered.

## 3 RESULTS

### 3.1 Loss metrics

For all models, the Attention U‐Net, HD U‐Net and 3D U‐Net, the training and validation loss curves demonstrated convergence (Figure S2). The Attention U‐Net validation loss curves showed a bigger spread between the 5‐folds compared to the validation loss curves of the HD U‐Net. The root mean square error (RMSE) and mean absolute error (MAE) loss for the Attention U‐Net and HD U‐Net show similar variability across all base models with and without addition of augmentation and/or extra input folds (Figure S3 and Figure S4). It took approximately 3 hours per fold to train the 2D U‐Nets and 24 hours per fold to train the 3D U‐Net, so approximately eight times more.

### 3.2 Clinical goals

**FIGURE 1:**
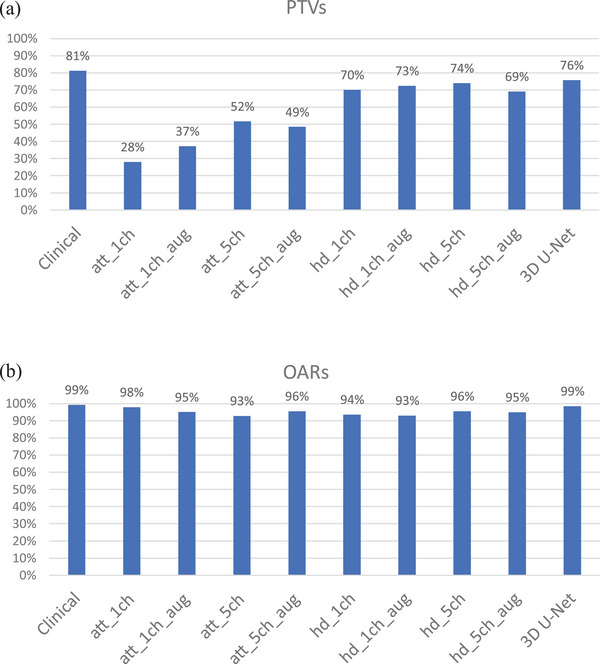
Bar‐chart of percentage of clinical goals reached. (a) Mean of clinical goals of all PTVs. (b) Mean of clinical goals of all OARs. Att: Attention U‐Net; HD: HD U‐Net; aug: with data augmentation; ch: number of input channels. OARs, organs at risk.

The performance of the Attention U‐Net and HD U‐Net and 3D U‐Net framework was assessed on meeting the clinical goals and compared to the clinical treatment plan (Figure 1). Overall, the HD U‐Net and 3D U‐Net outperformed the Attention U‐Net in meeting the clinical goals for the PTVs (Figure 1a). The PTVp average dose was in general too high in the Attention U‐Net (see also Table S2 and Table S3) which only improved slightly by the addition of channels or data augmentation. The slightly smaller percentage of clinical goals achieved using additional channels and augmentation compared to augmentation alone was caused mainly by a smaller number of patients for which the PTVp maximum dose goal was not reached. The percentage of PTV metrics fulfilled was between 69% and 74% for the HD U‐Net models while it was slightly higher with 76% and 81% for the 3D U‐Net and clinical plans, respectively. This was mainly caused by a somewhat too low PTVn1n2 D98% (e.g., D98% was on average still 93% (range 87%–97%) of the prescribed dose for the five channel HD U‐Net with data augmentation, but only 3/18 were above 95%. As the percentage of OAR clinical goals reached was already high for the one channel U‐Nets without data augmentation, adding channels or augmentation did not result in further improvements.

### 3.3 Dose volume criteria for primary target volumes

**FIGURE 2:**
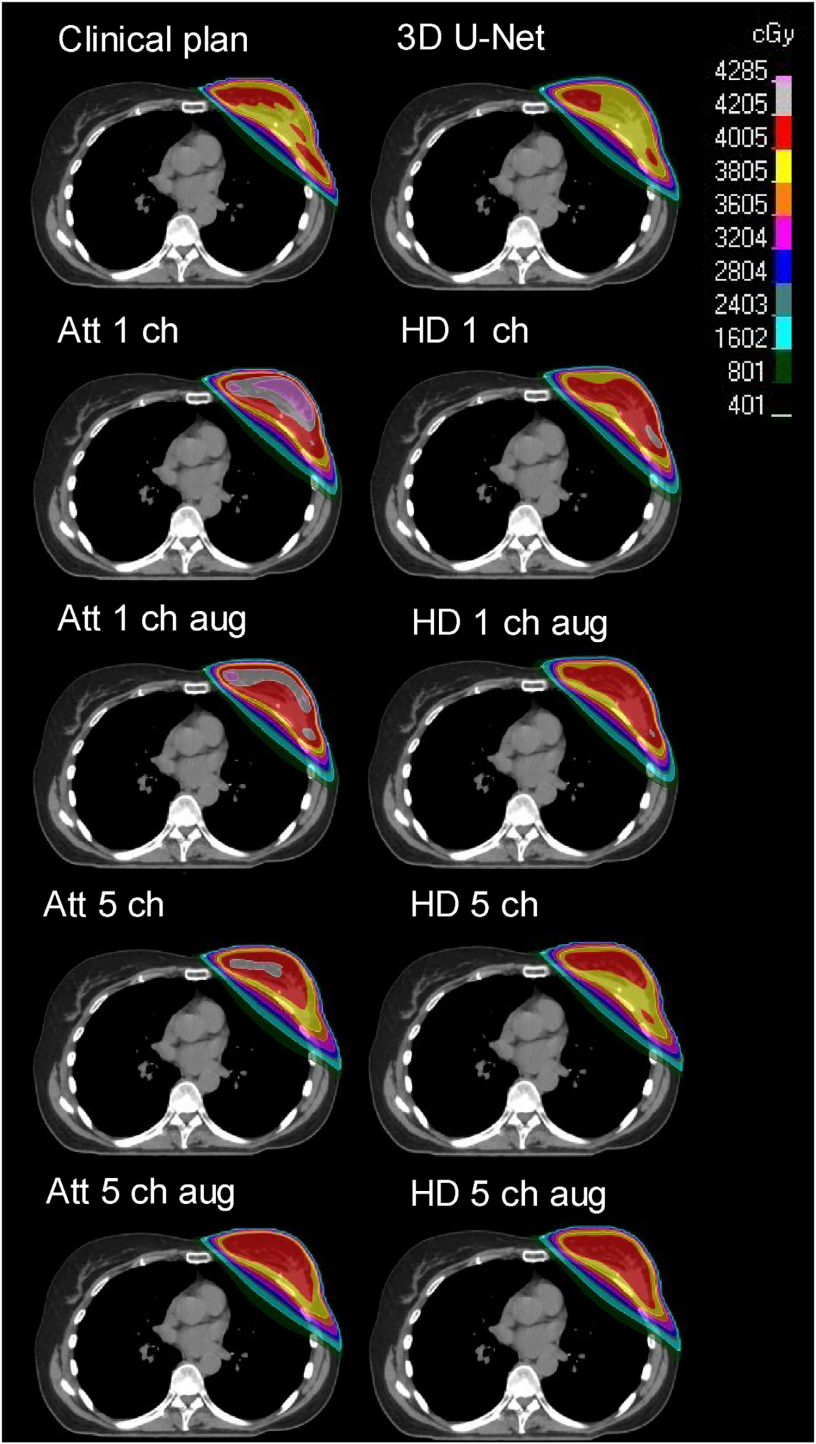
Example of dose predictions from all trained models and the clinical plan in an axial CT slice of one patient.

**FIGURE 3:**
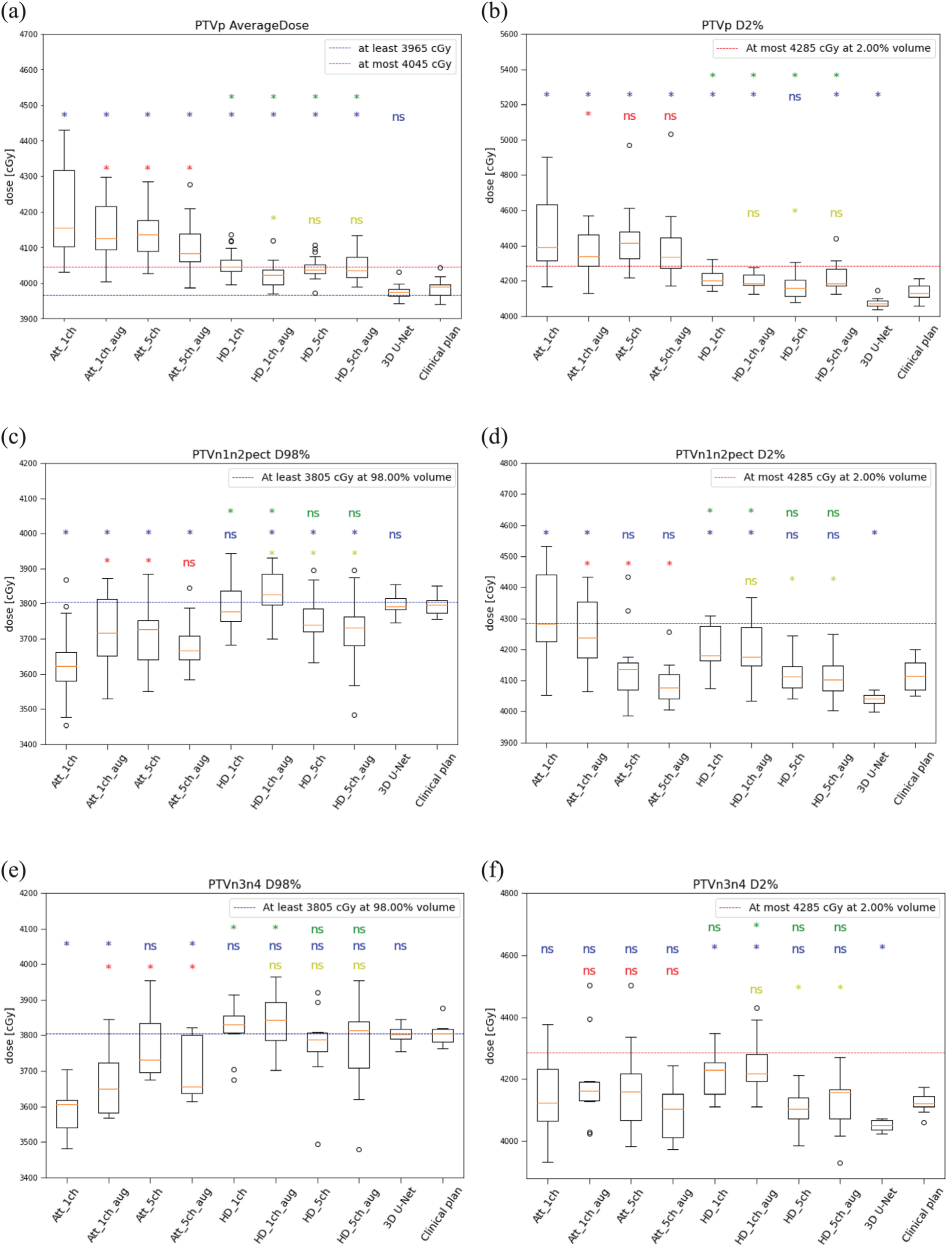
Boxplots of primary target dose volume clinical goals. Yellow lines represents the median values. (a) PTVp average dose (*n* = 18), (b) PTVp D2% (*n* = 18), (c) PTVn1n2 D98% (*n* = 18), (d) PTVn1n2 D2% (*n* = 18), (e) PTVn3n4 D98% (*n* = 10), (f) PTVn3n4 D2% (*n* = 10). Att: Attention U‐Net; HD: HD U‐Net; aug: with data augmentation; ch: number of input channels; blue annotation: statistical comparisons to clinical plan; red annotations: statistical comparison of Att with augmentation and/or extra input to the Att with only one channel; yellow annotations: statistical comparison of HD with augmentation and/or extra input to the HD with only one channel and green annotation: statistical comparison of Att to the HD counterparts with/without augmentation and/or extra input (Wilcoxon signed rank sum test; *: statistically significant *p* < 0.05; ns: not statistically significant *p* > 0.05).

As an example, Figure 2 presents dose distributions of one patient for all DL models trained in this study. Figure 3 shows boxplots of the DVH parameters of PTVs for all trained models.

When comparing the Attention U‐Net, HD U‐Net and 3D U‐Net to the clinical plan (blue annotations in Figure 3), significant differences were observed for the PTVp and PTVn1n2 metrics for 26 out of the 36 statistical tests (Figure 3a–d). However, for PTVn3n4, statistically significant differences were only found for 6 out of the 18 tests performed (Figure 3e,f). Looking somewhat closer at the comparison of the 3D U‐Net to the clinical plan, it can be seen that The 3D U‐Net showed no significant differences for the PTV average and nodes D98% (Figure 3a,c,e) but did show statistically significant lower max doses (Figure 3b,d,f).

When looking at the addition of extra input channels and/or data augmentation (thus 3 possible combinations), it can be seen that PTV dose metrics of the Attention U‐Net (red annotations) or HD U‐Net (yellow annotations) base models significantly improved in 12 out of the 18 tests and 9 out of the 18 tests performed, respectively.

Finally, the Attention U‐Net and HD U‐Net counterparts were compared on addition of extra input channels and/or data augmentation (green annotations in Figure 3). For PTVp the HD U‐Net in all cases showed statistically significant improvement over the Attention U‐Net (Figure 3a,b). The HD U‐Net with one channel with or without data augmentation was in 7 out of 8 tests better than its Attention U‐Net counterpart for the 8 metrics for the PTVn1n2 and PTVn3n4 volumes (Figure 3c,f). However, adding extra input channels or extra input channels with data augmentations showed no significant difference between the Attention U‐Net and HD U‐Net (Figure 3c,f).

### 3.4 Dose volume criteria for organs at risk

**FIGURE 4:**
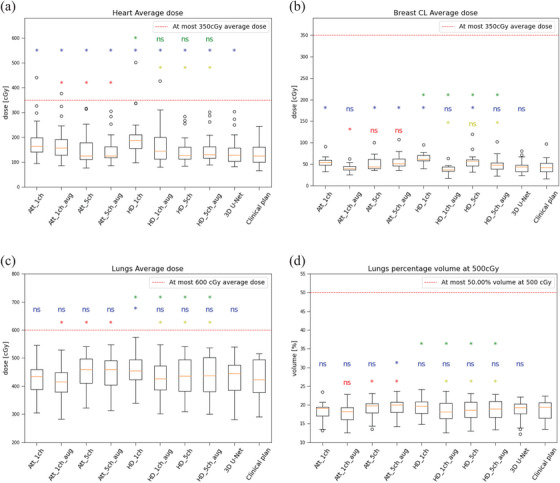
Boxplots of heart, contralateral breast and lungs average doses and lungs percentage volume above 5 Gy. (a) Heart average dose (*n* = 18), (b) Contralateral breast average dose (*n* = 16), (c) Lung average dose (*n* = 18), (d) Lung percentage volume above 5 Gy dose (*n* = 18). Annotations identical to Figure 3.

Figure 4 shows boxplots of the DVH parameters of the heart, lungs, and contralateral breast for all trained models while data for the external, humerus, and thyroid are given in Figure S5. When comparing the Attention U‐Net, HD U‐Net, and 3D U‐Net to the clinical plan (blue annotations), the clinical plan average heart dose metric was always statistically significantly better, although this was on average only 0.1 to 0.7 Gy for the different models. Five out of 10 tests were significant for the contralateral breast dose, while again the differences were very small with 0.0 to 0.2 Gy on average (see also Table S3). Only 9 out of the other 40 metrics for lungs, external, humerus, and thyroid were significant. Looking somewhat closer at the comparison of the 3D U‐Net to the clinical plan, only the heart and external body dose metrics were statistically significantly higher, but at a clinically insignificant level.

Overall, for all OARs the 3D U‐Net showed no significant difference to the clinical plan except for the average heart and external dose (Figure 4a and Figure S5).

When assessing addition of extra input channels and/or data augmentation for the OARs to either Attention U‐Net (red annotations) or HD U‐Net (yellow annotations) base models, it was found to improve predictions significantly for 9 out of the 15 test for both base models.

Finally, the Attention U‐Net and HD U‐Net counterparts were compared (green annotations in Figure 4 and Figure S5). When comparing the base model counterparts, we observed a preference for the Attention U‐Net for the most important OARs heart, lung contralateral breast and external but not for the humerus and thyroid. When adding either data augmentation or extra channels or both a slight preference for the HD U‐Net was observed in general.

## 4 DISCUSSIONS

In this study, we trained both 2D and 3D U‐Net models and evaluated the impact of data augmentation and the inclusion of extra channels on the overall performance and in meeting clinical goals. Our findings indicate that, overall, the 2D HD U‐Net exhibited superior performance compared to the 2D Attention U‐Net counterparts. Moreover, a comparative analysis between the HD U‐Net and Attention U‐Net to the 3D U‐Net revealed that the HD U‐Net demonstrated similar performance to the 3D U‐Net. The implementation of data augmentation and/or additional input channels led to the most significant performance enhancement for the PTVs of the Attention U‐Net, whereas less pronounced improvements were observed for the PTVs of the HD U‐Net.

Several studies have exploited various DL architectures to predict dose distributions. Osman et al. and Wu et al. compared multiple 3D U‐Net architectures, including the HD U‐Net and Attention U‐Net, for predicting dose distributions of 340 H&N plans and 261 cervical cancer, respectively.^12^, ^13^ Osman et al. found that all architectures exhibited similar performance, with minor differences for the OARs. Wu et al. also reported comparable performance across all architectures, with their 3D U‐Net showing best performance for the voxel‐based analysis. Both studies did include all OARs combined with the PTV and did not use data augmentation for training. When comparing our study to these previous studies, the Attention U‐Net and HD U‐Net, including all OARs, show differences in performance for the PTVs (Figure 1a and marginal differences in performance for the OARs; Figure 1b). The difference in PTVs could be due to training with a smaller dataset. However, adding data augmentation to overcome dataset size limitations did not improve performance in PTVs dose prediction. Therefore, we expect these differences are due to training the architectures in 2D instead of 3D.

In addition to comparing 3D DL architectures, Gronberg et al. reported on data augmentation techniques on dose predictions for 340 H&N cancer plans.^17^ Their best‐scoring model, a 3D dense dilated U‐Net, did not benefit from adding data augmentation. Unfortunately, they did not report if there was a benefit of adding data augmentation to the HD U‐Net they trained.

At last, Gu et al. utilized a different architecture, a 3D GAN, to predict dose prediction for H&N cancer, to investigate the influence of input channels on performance.^18^ Their study concluded that adding extra OARs as input did not result in a significant difference compared to only training with CT scans and PTV as input. This finding is in line with our results for the HD U‐Net. However, for the Attention U‐Net we observed a significant improvement in performance for the PTVs when adding extra input channels.

Although recent studies investigating dose predictions using DL architectures have predominantly trained 3D model, we deliberately chose to train our Attention and HD U‐Net models in 2D. This approach allowed us to evaluate whether 2D DL models are competitive to 3D models. Additionally, we incorporated distance maps to provide geometrical information. Training our 2D models reduced the training time approximately 8 fold, which makes the threshold to retrain models with new data lower and saves computational costs and energy. One might consider using a patch‐based approach during training to further reduce computational costs.

A limitation we acknowledge in our study is our limited dataset, consisting of left‐sided breast cancer patients with either lymph node levels 1 and 2 or 1 up to 4. By segregating these two groups and conducting a more precise analysis we might have identified subtle differences in the results for the PTVs and OARs. Also, we used a single institution dataset, meaning we used one specific treatment technique. As such, the results can only be used directly in other institutions if they use the same technique, although we used national evaluation criteria to evaluate our results. Furthermore, we did not integrate dose mimicking to create clinically deliverable dose distributions. We expect that mimicking would further reduce the differences between models as was reported previously.^6^ Therefore, mimicking would limit the possibility of observing the impact of adding data augmentation and/or extra input.

## 5 CONCLUSION

In conclusion, we trained three DL models to predict dose distributions for locally advanced left‐sided breast cancer radiotherapy. Our findings indicate that the 2D HD U‐Net, with additional input channels and/or data augmentation, performed comparable to the 3D commercially available U‐Net. Furthermore, the addition of data augmentation and/or extra input channels significantly enhanced performance for the Attention U‐Net.

## AUTHOR CONTRIBUTIONS

All authors meet the author criteria as set forth in the authorship criteria of the JACMP. Nienke L. M. Bakx was supported by a grant from RaySearch, Stockholm, Sweden. RaySearch had no role in the data collection, analysis, or publication of this study.

## CONFLICT OF INTEREST STATEMENT

The authors have nothing to report.

## Supporting information



Supporting Information

Supporting Information

Supporting Information

Supporting Information

Supporting Information

Supporting Information

Supporting Information

Supporting Information

## Data Availability

The data that support the findings of this study are available from the corresponding author upon reasonable request.
