# Supplementary material for: A comparative analysis of deep learning architectures with data augmentation and multichannel input for locoregional breast cancer radiotherapy
Source: J Appl Clin Med Phys. 2025 Feb 20;26(6):e70047. doi: 10.1002/acm2.70047 (PMC12148752; doi:10.1002/acm2.70047)
Supplement: Supplementary file 1 — Supporting Information [file ACM2-26-e70047-s002.docx]

| (a)  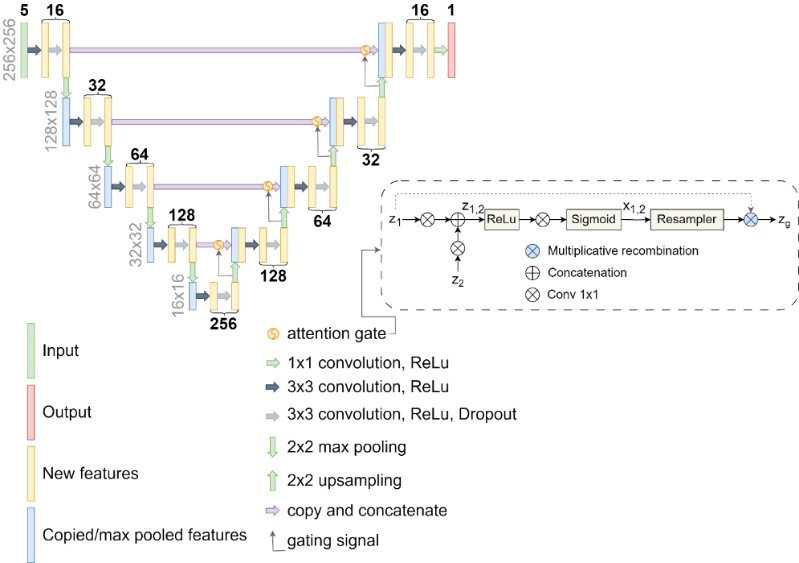 | (b)  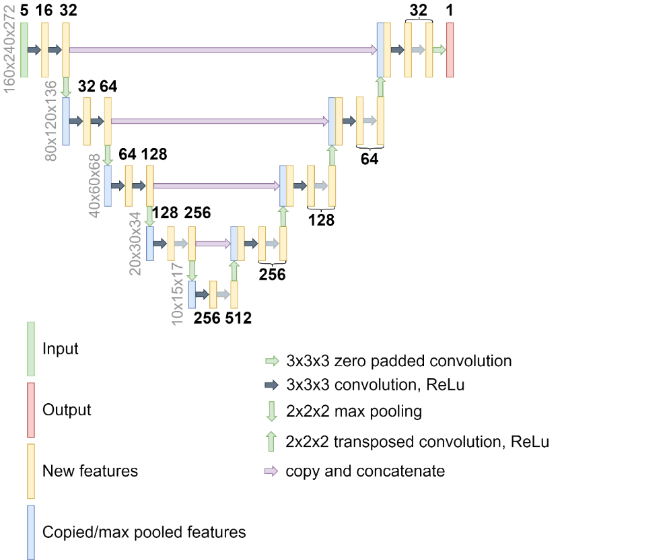 |
| --- | --- |
| (c)  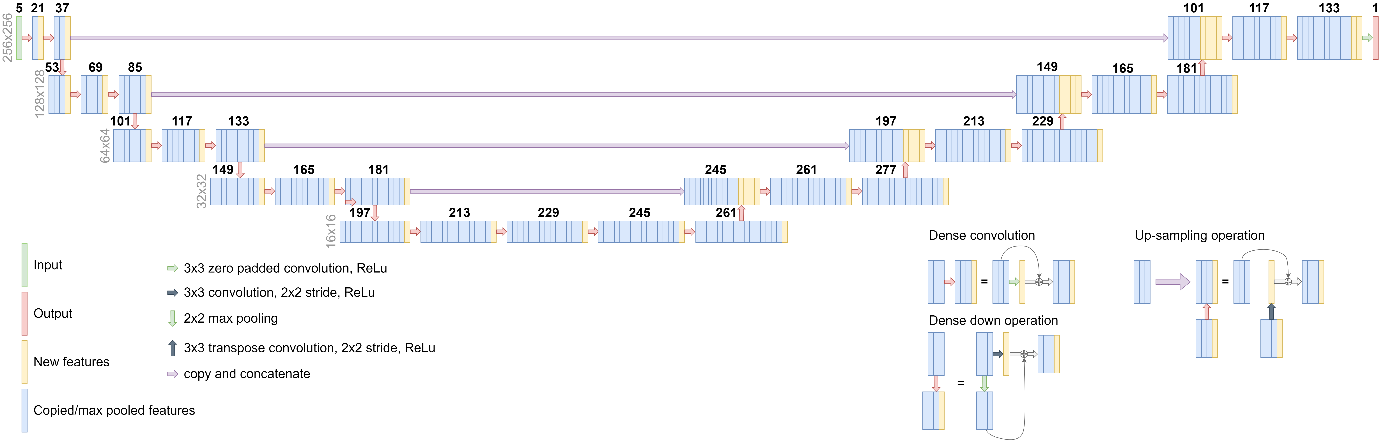 | |
| **FIGURE S1** Schematic representation of the three architectures investigated in this study  (a) Attention U-Net, (b) 3D U-Net and (c) HD U-Net. For the 3D model, the input is centered around the PTV, allowing it to be cropped and thus have a smaller x and y dimension while still containing all relevant information. For the 2D input, it was not chosen to center or crop the input, but to pad all input to 256 x 256 to have an equal dimension on all patients. The axial resolution is not different across the different models, since no further pre-processing was used other than cropping, centering or padding. | |
