# Supplementary material for: A comparative analysis of deep learning architectures with data augmentation and multichannel input for locoregional breast cancer radiotherapy
Source: J Appl Clin Med Phys. 2025 Feb 20;26(6):e70047. doi: 10.1002/acm2.70047 (PMC12148752; doi:10.1002/acm2.70047)
Supplement: Supplementary file 3 — Supporting Information [file ACM2-26-e70047-s003.docx]

| 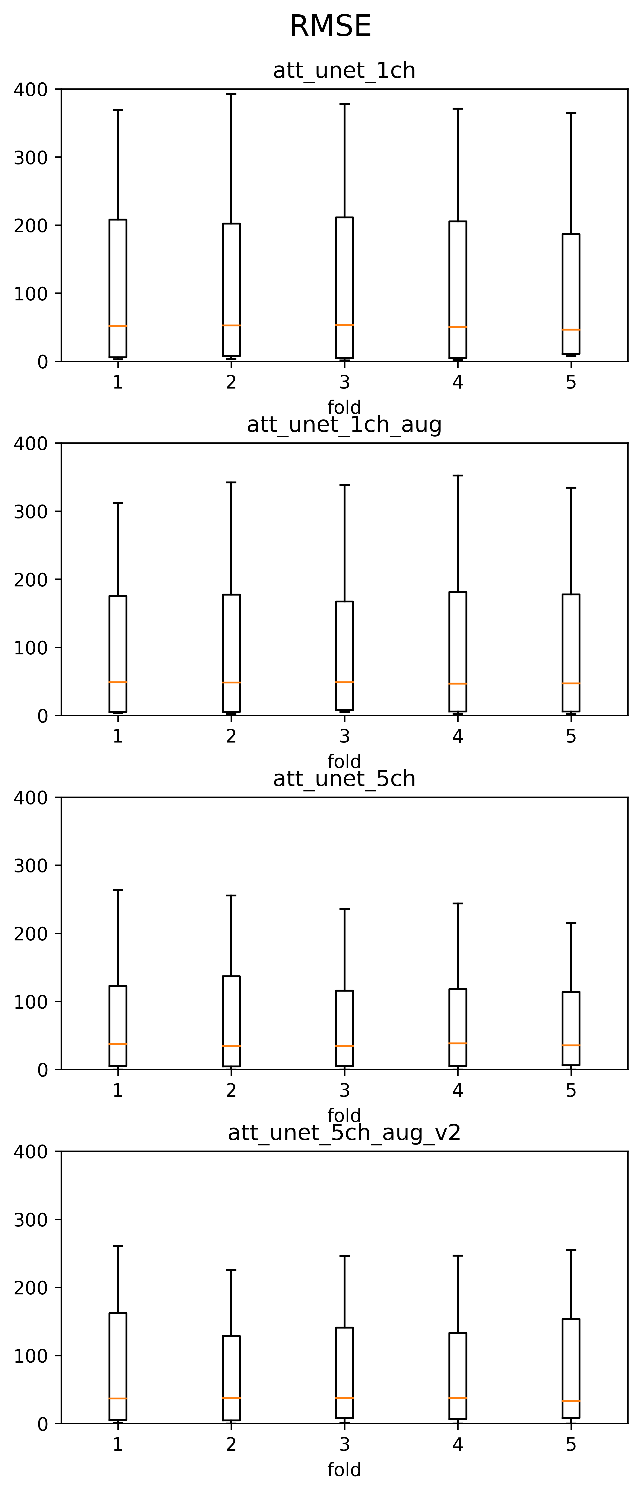 | 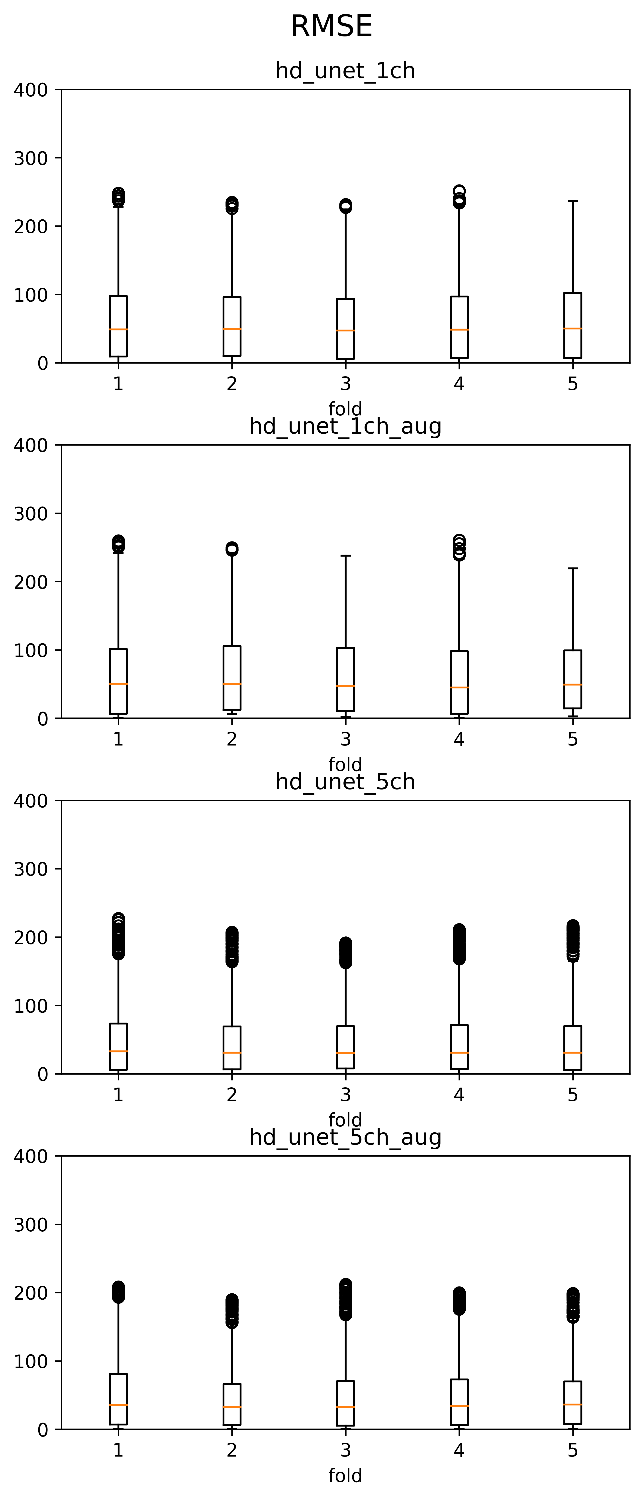 |
| --- | --- |
| **FIGURE S3** Box plots of RMSE loss metrics. (a) Attention U-Net, (b) HD U-Net. The yellow lines indicate the median values. | |
