# Supplementary material for: A comparative analysis of deep learning architectures with data augmentation and multichannel input for locoregional breast cancer radiotherapy
Source: J Appl Clin Med Phys. 2025 Feb 20;26(6):e70047. doi: 10.1002/acm2.70047 (PMC12148752; doi:10.1002/acm2.70047)
Supplement: Supplementary file 5 — Supporting Information [file ACM2-26-e70047-s005.docx]

| (a)  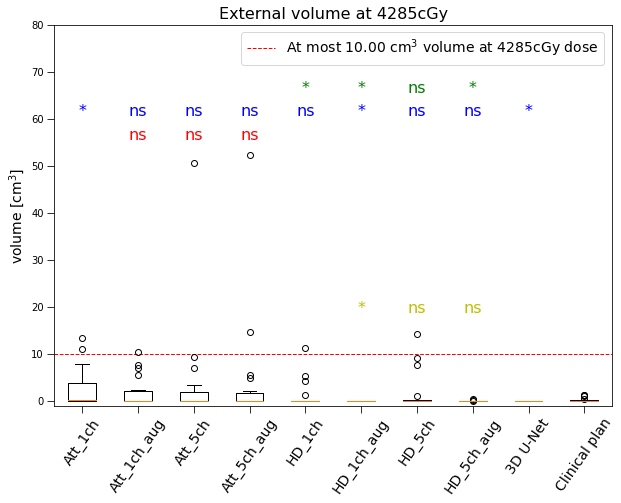 | (b)  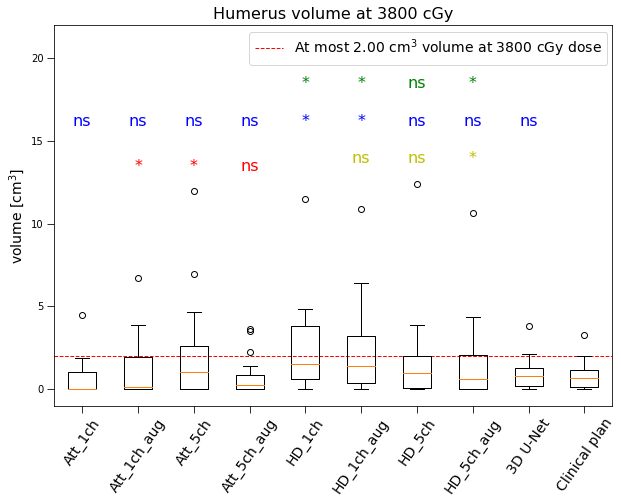 |
| --- | --- |
| (c)  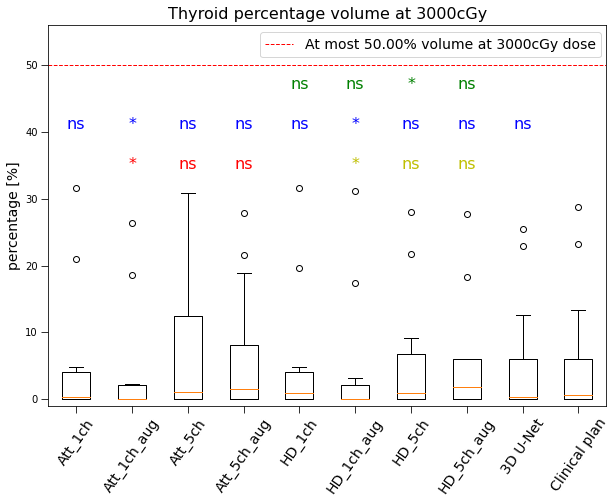 |  |
| **FIGURE S5** Boxplots of (a) External-PTV V_4285Gy_ (n=18), (b) Humerus_PRV10 V_38Gy_ (n=18) and (c) Thyroid V_30Gy_ (n=13). Att; Attention U-Net, HD: HD U-Net, aug: with data augmentation, ch: number of input channels, blue annotation: statistical comparisons to clinical plan, red annotations: statistical comparison of Att with augmentation and/or extra input to the Att with only one channel, yellow annotations: statistical comparison of HD with augmentation and/or extra input to the HD with only one channel and green annotation: statistical comparison of Att to the HD counterparts with/without augmentation and/or extra input. (Wilcoxon signed rank sum test, *: statistically significant P<0.05, ns: not statistically significant P>0.05.) | |
