# Supplementary material for: A comparative analysis of deep learning architectures with data augmentation and multichannel input for locoregional breast cancer radiotherapy
Source: J Appl Clin Med Phys. 2025 Feb 20;26(6):e70047. doi: 10.1002/acm2.70047 (PMC12148752; doi:10.1002/acm2.70047)
Supplement: Supplementary file 6 — Supporting Information [file ACM2-26-e70047-s008.docx]

**TABLE S1** Overview of all clinical goals based on Hurkmans et al. (1).

| ROI | Description | Goal |
| --- | --- | --- |
| PTVp | PTV of the whole left breast cropped 5 mm inside external | D­_98%_ ≥ 38.0 Gy |
|  |  | D_2%_ ≤ 42.8 Gy |
|  |  | 39.6 Gy ≤ D_average_ ≤ 40.5 Gy |
| PTVn1n2 | PTV of lymph nodes level 1&2 cropped 5 mm inside external | D_98%_ ≥ 38.0 Gy |
|  |  | D_2%_ ≤ 42.9 Gy |
| PTVn3n4 | PTV of lymph nodes level 3&4 cropped 5 mm inside external | D_98%_ ≥ 38.0 Gy |
|  |  | D_2%_ ≤ 42.9 Gy |
| Heart | Heart | D_mean_ ≤ 2.5 Gy |
| Breast CL | Contralateral breast | D_average_ ≤ 1 Gy |
| Lungs | Both lungs combined | D_average_ ≤ 6.0 Gy |
|  |  | V_5Gy_ ≤ 50% |
| External-PTV | Full patient body, without all PTVs | V_42.8Gy_ ≤ 10 cm^3^ |
| Thyroid | Thyroid | V_30Gy_ ≤ 50% |
| Esophagus | Esophagus | V_30Gy_ ≤ 5% |
