# Supplementary material for: A comparative analysis of deep learning architectures with data augmentation and multichannel input for locoregional breast cancer radiotherapy
Source: J Appl Clin Med Phys. 2025 Feb 20;26(6):e70047. doi: 10.1002/acm2.70047 (PMC12148752; doi:10.1002/acm2.70047)
Supplement: Supplementary file 7 — Supporting Information [file ACM2-26-e70047-s004.docx]

**TABLE S2** Overview all models and total amount of patients that meet the clinical goals for the PTVs and Heart, Lungs and Breast CL.

|  | **PTVp average dose** | **PTVp D_2%_** | **PTVn1n2 D_98%_** | **PTVn1n2 D_2%_** | **PTVn3n4 D_98%_** | **PTVn3n4 D_2%_** | **Heart average dose** | **Breast CL average dose** | **Lungs average dose** | **Lungs V_5Gy_** |
| --- | --- | --- | --- | --- | --- | --- | --- | --- | --- | --- |
| Amount of  patients tested | 18 | 18 | 18 | 18 | 10 | 10 | 18 | 16 | 18 | 18 |
| Clinical | 16 | 18 | 8 | 18 | 6 | 10 | 18 | 16 | 18 | 18 |
| Att_1ch | 2 | 3 | 1 | 9 | 0 | 8 | 17 | 15 | 18 | 18 |
| Att_1ch_aug | 2 | 5 | 5 | 12 | 1 | 8 | 17 | 15 | 18 | 18 |
| Att_5ch | 2 | 15 | 3 | 16 | 3 | 8 | 18 | 14 | 18 | 18 |
| Att_5ch_aug | 4 | 6 | 1 | 18 | 3 | 10 | 18 | 14 | 18 | 18 |
| HD_1ch | 9 | 15 | 7 | 16 | 8 | 8 | 17 | 15 | 18 | 18 |
| HD_1ch_aug | 12 | 18 | 11 | 14 | 6 | 7 | 17 | 15 | 18 | 18 |
| HD_5ch | 15 | 17 | 3 | 18 | 5 | 10 | 18 | 14 | 18 | 18 |
| HD_5ch_aug | 10 | 15 | 3 | 18 | 6 | 10 | 18 | 14 | 18 | 16 |
| 3D U-Net | 13 | 18 | 6 | 18 | 5 | 10 | 18 | 15 | 18 | 18 |
