# Supplementary material for: A comparative analysis of deep learning architectures with data augmentation and multichannel input for locoregional breast cancer radiotherapy
Source: J Appl Clin Med Phys. 2025 Feb 20;26(6):e70047. doi: 10.1002/acm2.70047 (PMC12148752; doi:10.1002/acm2.70047)
Supplement: Supplementary file 8 — Supporting Information [file ACM2-26-e70047-s001.docx]

**TABLE S3** Average dose and standard deviation for PTVp, heart, lungs and contralateral breast.

|  | PTVp | Heart | Lungs | Breast CL |
| --- | --- | --- | --- | --- |
|  | Dose [Gy] | Dose [Gy] | Dose [Gy] | Dose [Gy] |
| Clinical | 39.9 ± 0.2 | 1.4 ± 0.5 | 4.3 ± 0.7 | 0.4 ± 0.2 |
| Att_1ch | 42.0 ± 1.3 | 1.9 ± 0.9 | 4.2 ± 0.6 | 0.6 ± 0.1 |
| Att_1ch_aug | 41.5 ± 0.8 | 1.8 ± 0.8 | 4.1 ± 0.7 | 0.4 ± 0.1 |
| Att_5ch | 41.4 ± 0.7 | 1.6 ± 0.7 | 4.4 ± 0.7 | 0.5 ± 0.2 |
| Att_5ch_aug | 41.0 ± 0.7 | 1.6 ± 0.6 | 4.4 ± 0.7 | 0.6 ± 0.2 |
| HD_1ch | 40.5 ± 0.4 | 2.1 ± 0.9 | 4.5 ± 0.6 | 0.6 ± 0.1 |
| HD_1ch_aug | 40.4 ± 0.3 | 1.8 ± 0.9 | 4.2 ± 0.7 | 0.4 ± 0.1 |
| HD_5ch | 40.2 ± 0.4 | 1.5 ± 0.6 | 4.3 ± 0.7 | 0.6 ± 0.2 |
| HD_5ch_aug | 40.5 ± 0.4 | 1.6 ± 0.6 | 4.3 ± 0.7 | 0.5 ± 0.2 |
| 3D U-Net | 39.7 ± 0.2 | 1.5 ± 0.6 | 4.3 ± 0.7 | 0.4 ± 0.2 |
